# p53: key conductor of all anti-acne therapies

**DOI:** 10.1186/s12967-017-1297-2

**Published:** 2017-09-19

**Authors:** Bodo C. Melnik

**Affiliations:** Department of Dermatology, Environmental Medicine and Health Theory, University of Osnabrück, Am Finkenhügel 7a, 49076 Osnabrück, Germany

**Keywords:** Acne therapy, Apoptosis, Immortalized sebocytes, p53, SV40, TRAIL

## Abstract

This review based on translational research predicts that the transcription factor p53 is the key effector of all anti-acne therapies. *All*-*trans* retinoic acid (ATRA) and isotretinoin (13-*cis* retinoic acid) enhance p53 expression. Tetracyclines and macrolides via inhibiting p450 enzymes attenuate ATRA degradation, thereby increase p53. Benzoyl peroxide and hydrogen peroxide elicit oxidative stress, which upregulates p53. Azelaic acid leads to mitochondrial damage associated with increased release of reactive oxygen species inducing p53. p53 inhibits the expression of androgen receptor and IGF-1 receptor, and induces the expression of IGF binding protein 3. p53 induces FoxO1, FoxO3, p21 and sestrin 1, sestrin 2, and tumour necrosis factor-related apoptosis-inducing ligand (TRAIL), the key inducer of isotretinoin-mediated sebocyte apoptosis explaining isotretinoin’s sebum-suppressive effect. Anti-androgens attenuate the expression of miRNA-125b, a key negative regulator of p53. It can thus be concluded that all anti-acne therapies have a common mode of action, i.e., upregulation of the guardian of the genome p53. Immortalized p53-inactivated sebocyte cultures are unfortunate models for studying acne pathogenesis and treatment.

## Background

Acne vulgaris is the most common inflammatory skin disease affecting more that 80% of adolescents of developed countries [1]. Four major factors are involved in acne pathogenesis: (1) increased sebum production, (2) hyperkolonization and biofilm formation of *Propionibacterium acnes* (*P. acnes*), (3) increased acroinfundibular keratinocyte proliferation with comedo formation, (4) and follicular as well as perifollicular inflammation [2]. Sebum is the secretory product of holocrine secretion of sebocytes derived from sebaceous glands (SGs) [3]. Excessive production of sebum containing higher amounts of monounsaturated pro-inflammatory lipids results from exaggerated sebocyte activity, which is induced by increased insulin-like growth factor-1 (IGF-1) and androgen signalling [2]. Recent evidence underlines that dietary factors, especially hyperglycaemic carbohydrates and milk consumption, increase insulin/IGF-1 signalling promoting acne [4–7]. Enhanced activity of the phosphoinositide-3-kinase (PI3K)/AKT pathway down-regulates the nuclear activity of the metabolic transcription factor FoxO1 [8–11], the transcription factor of starvation [12]. Acne is associated with increased activity of mechanistic target of rapamycin complex 1 (mTORC1) [13, 14], which promotes the expression of two lipogenic transcription factors, sterol regulatory element binding protein-1c (SREBP1c) and peroxisome proliferator-activated receptor-γ (PPARγ) [15]. It has been predicted that mTORC1 is activated in the skin of acne patients [16], which has been confirmed experimentally [10, 17]. SREBP1, which is upregulated via increased AKT/mTORC1 signalling plays a key role in sebaceous lipogenesis [18, 19], and in addition induces sebum fatty acid monounsaturation [20, 21], that plays a crucial role in comedogenesis and inflammation of acne [22, 23]. Activated IGF-1/mTORC1 signalling promotes the expression of the anti-apoptotic protein survivin [24, 25], which has recently been found to be upregulated in the skin of acne patients [26]. Intriguingly, serum IGF-1 levels of acne patients significantly correlate with survivin expression [26]. Morphologically, acne vulgaris is characterized by SG hyperplasia with increased production of sebum with higher amounts of pro-inflammatory and comedogenic monounsaturated fatty acids [22]. Increased IGF-1 signalling of puberty superimposed with insulin signalling of Western diet (hyperglycaemic carbohydrates and milk) provide the input signals for disturbed acne metabolomics including mTORC1-S6K1-mediated insulin resistance [22, 27, 28].

It is the intention of this paper to demonstrate that all these acne-related deviations of molecular signalling will be corrected by increasing the expression of the key transcription factor p53, known as the guardian of the human genome [29, 30]. Translational evidence will be presented showing that all common anti-acne therapies used in today’s clinical practice converge in upregulating the expression of p53.

## Retinoic acid

*All*-*trans* retinoic acid (ATRA), the prototype of topical retinoids, is comedolytic, resolves precursor microcomedones and is anti-inflammatory [31, 32]. Topical ATRA has been shown to transform the horn-filled utriculi of the *rhino mouse* into normal follicles [33]. ATRA-mediated upregulation of p53 has been reported in several cells including cervical carcinoma cells, acute myeloblastic leukaemia cells, human embryo carcinoma cells, and glioma cells [34–37]. In human embryonic stem cells, ATRA-mediated upregulation of CBP/p300 acetylated p53 at lysine 373, which leads to p53 dissociation from E3-ubiquitin ligases HDM2 and TRIM24, thereby stabilizing p53 expression [38]. It has been demonstrated in primary human epidermal keratinocytes that ATRA regulates many genes associated with cell cycle arrest and programmed cell death [39]. In human keratinocytes, ATRA increases the expression of p53, pro-apoptotic caspases, and sensitizes keratinocytes to apoptosis [40]. Chronic activation of p53 in mice resulted in the loss of SGs associated with a depletion of B-lymphocyte-induced nuclear maturation protein 1 (BLIMP1) positive SG cells explained by suppressed mTORC1 activity [41]. In fact, two p53 target genes, *SESN1* and *SESN2*, activate the AMP-responsive protein kinase (AMPK) and target it to phosphorylate TSC2 and stimulate its GAP activity, thereby inhibiting mTORC1 [42, 43].

p53 intersects at multiple points with the regulation of inflammation [44]. The pro-inflammatory transcription factor nuclear factor κB (NFκB) and p53 can act as functional antagonists. The E3 ubiquitin ligase *mouse double minute homolog 2* (MDM2), whose gene is transcriptionally activated by p53, can act as a direct negative regulator of NFκB by binding and inhibiting p65RelA [45]. Furthermore, ATRA-p53-induced neutrophil apoptosis may reduce inflammation in acne [46–48]. ATRA has also been shown to induce apoptosis and suppression of NFκB signalling in adult T cell leukaemia cells [49]. Both, ATRA-p53-induced inhibition of NFκB and neutrophil as well as T cell apoptosis may contribute to the anti-inflammatory effect of ATRA in the treatment of acne.

## Peroxides and photodynamic therapy

Benzoyl peroxide (BPO) is an anti-bacterial topical agent that kills *P. acnes* through the release of free oxygen radicals and is also mildly keratolytic and comedolytic [50–52]. BPO treatment decreased the size of gold hamster ear SGs and the number of sebocytes entering the S-phase of the cell cycle [53]. The mitotic index of BPO-treated sebocytes exhibited a reduction of 33.8% [53]. Similar results have been obtained in autoradiographic studies of human SGs [54, 55]. Although less efficient than ATRA, BPO decreased the size and numbers of corneocytes [56], and reduced comedo formation in the rabbit ear microcomedo prevention assay [57]. HaCaT keratinocytes incubated for 24 h with BPO exhibited a dose-dependent cytotoxicity at concentrations above 250 μm. It is important to mention that BPO is a potent inducer of oxidative stress increasing the intracellular ratio of oxidized to reduced glutathione (GSSG/GSH) in treated keratinocytes [58]. Notably, BPO interacts with mitochondria, inhibits mitochondrial respiration and induces mitochondrial swelling [59]. In a comparable manner, topical hydrogen peroxide (H_2_O_2_) treatment reduced the number of inflammatory and non-inflammatory acne lesions [60–62]. It has been demonstrated in C2C12 muscle cells that H_2_O_2_ induced mitochondrial permeability transition pore opening and p53 activation. Intriguingly, testosterone treatment prior to H_2_O_2_ administration reduced p53 activation and prevented mitochondrial permeability transition pore opening [63]. After mitochondrial damage, p53 maintains the mitochondrial genome through its translocation into mitochondria and interactions with mitochondrial DNA repair proteins. This mechanism provides a further explanation for the upregulation of p53 after mitochondrial insults such as challenges with BPO or H_2_O_2_ [64]. Acting as a signal, H_2_O_2_ circumvents antioxidant defence by over-oxidizing peroxiredoxins, the enzymes that metabolize peroxides. Sestrins, a family of proteins whose expression is induced by p53, are required for regeneration of peroxiredoxins containing Cys-SO_2_H, thus re-establishing the antioxidant firewall [65]. Sestrins accumulate in cells exposed to oxidative stress, potentiate AMPK, which finally inhibits mTORC1 [66]. It is well appreciated that oxidative stress and mitochondrial damage-mediated generation of reactive oxygen species (ROS) promote an immediate p53 response [67]. Oxidative stress activates p53 and in turn inhibits cell proliferation and growth through induction of Sestrin 1 and Sestrin 2, which inhibit mTORC1 [67]. Remarkably, metformin, which as well exhibits beneficial effect in the treatment of acne [68], via activation of AMPK and inhibition of mTORC1 [69], operates on the same pathway as AMPK-activating peroxides. In fact, metformin has been shown to increase p53 expression in patients with polycystic ovary syndrome [70].

The major effect of photodynamic therapy (PDT) in acne is the generation of ROS [71, 72]. Thus, PDT mimics the effects of BPO-mediated upregulation of p53. It should thus be expected that retinoid- and BPO-mediated upregulation of p53 may exert synergistic effects in the treatment of acne. In fact, adapalene and BPO significantly decreased the expression of the proliferation marker Ki67, α_2_ and α_6_ integrins, TLR-2, β-defensin-4 and IL-8 in inflammatory acne skin, whereas single treatments with adapalene or BPO alone were less effective [73].

## Azelaic acid

Azelaic acid (AZA), a saturated C9-dicarboxylic acid, is mildly effective as a comedolytic, anti-bacterial, and anti-inflammatory topical agent for the treatment of acne vulgaris [74, 75]. In cultured keratinocytes, AZA exerted time- and dose-dependent anti-proliferative effects associated with an early marked swelling and damage of mitochondria [76–78]. AZA and other C8-C13 dicarboxylic acids inhibit mitochondrial respiration and promote mitochondrial damage [79]. It has been shown that phosphatidylcholine esterified with AZA induced mitochondrial apoptosis at low micromolar concentrations in various cell lines [80]. Isolated exposed mitochondria rapidly swelled and released cytochrome c and apoptosis-inducing factor [80]. Mitochondrial damage results in mitochondrial ROS production, which upregulates the expression of p53, which promotes mitochondria-mediated apoptosis [81]. In fact, it has recently been confirmed that AZA induces apoptosis in acute myeloid leukaemia cells in a dose-dependent manner [82]. Thus, AZA shares mechanistic similarities with peroxide-induced mitochondrial disturbances upregulating the p53 response.

## Tetracyclines and macrolides

Tetracyclines are considered the first-line therapy in moderate to severe acne [83]. Apart from their anti-bacterial activities against *P. acnes* and bacterial lipases, non-antibiotic properties of tetracyclines gained recent attention [84]. The observation that sub-antimicrobial dosing of doxycycline showed equal efficacy as conventional anti-bacterial doxycycline treatment of inflammatory lesions in moderate and severe acne underlined the importance of tetracyclines’ non-antibiotic effects in acne [85]. Tetracylines, hypervitaminosis A, and systemic isotretinoin treatment share an increased risk for *pseudotumor cerebri,* which already points to a common underlying pathogenic mode of action [86]. ATRA homeostasis in the adult CNS is tightly controlled through local ATRA synthesis and cytochrome P450 (CYP450)-mediated inactivation of ATRA [87]. In neuronal cells, minocycline increased ATRA levels via inhibiting p450-mediated ATRA degradation [87]. This observation prompted Hellmann-Regen et al. [88] to speculate that tetracyclines and erythromycin may exert their pharmacological mode of action in acne via suppression of p450-mediated ATRA degradation in the skin. In fact, these investigators provided experimental evidence that minocycline potently blocked ATRA degradation in rat skin microsomes, and strikingly enhanced ATRA levels in ATRA-synthesizing cell cultures in a dose-dependent manner [89]. Several studies underline that tetracyclines and macrolides such as erythromycin and azithromycin suppress ATRA-catabolizing p450 enzymes modifying cellular ATRA homeostasis [90–92]. Intracellular upregulation of ATRA is thus the common denominator of p450-inhibiting agents that finally upregulate p53. A link between p450-regulation and mTORC1 signalling has recently been suggested [93]. In fact, it has been demonstrated that minocycline upregulated p53 and inactivated the AKT/mTORC1 pathway [94].

In contrast, it should be expected that p450-inducing agents such as isoniazid, phenobarbital, rifampicin, phenytoin, glucocorticosteroids and others [95–97] may lower intracellular ATRA levels and thus increase the risk for acne. Indeed, the majority of drugs reported to promote acne and acneiform drug eruption are p450-inducing agents [98–100].

## Oral isotretinoin

**Fig. 1:**
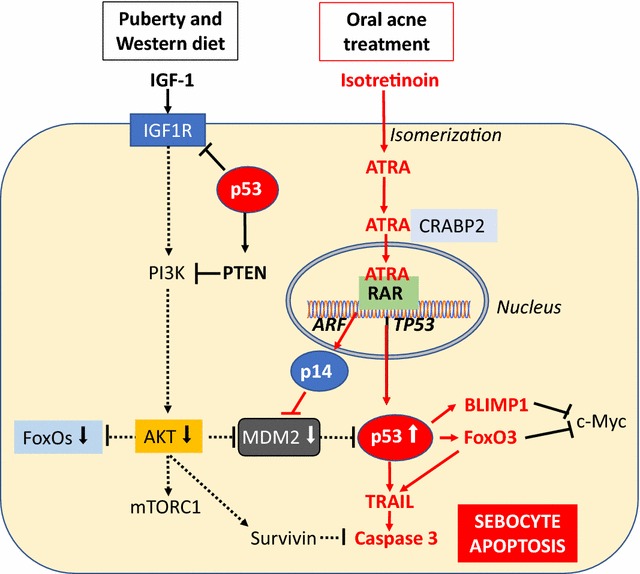
Isotretinoin-induced p53-mediated sebocyte apoptosis. In the sebocyte, isotretinoin is isomerized to *all*-*trans*-retinoic acid (ATRA), which is transported to the nucleus via cellular retinoic acid binding protein 2 (CRABP2). In the nucleus, ATRA binds to retinoic acid receptor (RAR) and activates RAR-responsive genes such as TP53, which promotes the expression of p53. ATRA-induced expression of ARF promotes the expression of p14, which is a negative regulator of mouse double minute 2 (MDM2), the key inhibitor of p53 via proteasomal degradation of p53. Increased IGF-1 signalling is attenuated by p53 and reduces the activity of the kinase AKT, that via phosphorylation inhibits the activity of FoxO1 and FoxO3 but stimulates MDM2. Thus, isotretinoin increases p53 activity via its direct transcriptional induction and posttranslational inhibition of its negative regulator MDM2. Subsequently, increased p53 activates several apoptosis-promoting proteins such as tumour necrosis factor-related apoptosis-inducing ligand (TRAIL). p53-attenuated IGF-1 signalling reduces the expression of survivin, a critical inhibitor of caspase 3. p53-induced expression of BLIMP1 and FoxO3 suppresses c-Myc, a key transcription factor of sebocyte differentiation. The final outcome is sebocyte apoptosis, the primary mechanism of isotretinoin-induced sebum suppression

Oral isotretinoin (13-*cis* retinoic acid), an isomer of ATRA, has been used for the treatment of severe recalcitrant acne for nearly four decades [101]. Its use has proven successful for most patients with severe acne, resulting in decreased sebum production and marked reduction of inflammatory lesions [102–105]. It is important to realize that the sebum-suppressive action of isotretinoin is not related to decreased lipid synthesis of individual sebocytes but is caused by sebocyte death, which histologically corresponds to the involution of SGs during isotretinoin treatment [106–108]. In pioneering histological and planimetrical studies, a marked decrease in the size of SGs of up to 90% of the pre-treatment values has been observed after 12 weeks of treatment. Additionally, the ratio of the differentiating pool of sebocytes versus the undifferentiating cell pool changed from 2:1 to 1:7 [107]. Furthermore, the labelling index of sebocytes regressed significantly under isotretinoin therapy. Today, this dramatic SG involution can be explained by isotretinoin-mediated sebocyte apoptosis (programmed cell death). Sebocytes are able to isomerize 13-*cis* retinoic acid to *all*-*trans* retinoic acid (ATRA), which binds to and activates retinoic acid receptors (RARs) that modify gene expression [109] (Fig. 1). One most important ATRA-responsive gene is the transcription factor p53 [39]. Activated p53 induces the expression of the pro-apoptotic effector TRAIL (tumour necrosis factor-related apoptosis-inducing ligand) [110]. There are two p53 DNA-binding sites in the human TRAIL promoter region [110]. Furthermore, ATRA induces RAR-dependent transcriptional upregulation of the TRAIL receptor 1 (TRAIL-R1, also known as death receptor 4) [111], thus promotes apoptotic TRAIL signalling at the ligand- and receptor level. ATRA also activates the expression of the transcription factor FoxO3a [112, 113]. p53 directly binds and activates the expression of the *FOXO3A* gene [114, 115]. Many of the genes targeted by p53 were also targeted by FOXO transcription factors, indicating that p53 functions in a coordinate manner to suppress gene expression downstream of PI3K/AKT/mTORC1 signalling [116, 117]. Both ATRA-induced p53 and ATRA-induced FoxO3a synergistically promote TRAIL expression [118]. In isotretinoin treated acne patients, TdT-mediated dUTP-biotin nick end labelling (TUNEL), a marker of apoptotic cells, was strongest in the nuclei of sebocytes in the basal layer and in early differentiated sebocytes adjacent to the basal layer of SGs [119]. In accordance, upregulated TRAIL expression has been observed in the basal and suprabasal layers of SG during isotretinoin treatment of acne patients [120], which allows the conclusion that isotretinoin-ATRA-p53/FoxO3a-induced TRAIL signalling explains isotretinoin-induced sebocyte apoptosis resulting in the involution of SGs (Fig. 1). Kelhälä et al. [106] confirmed increased TRAIL mRNA expression in lesional skin of isotretinoin-treated acne patients. TRAIL-mediated activation of caspase 8 and caspase 3 inactivates p63 [121], a critical marker of seboblasts/progenitor cells located in the outermost layer of SGs [122]. Thus, isotretinoin via increased p53 signalling apparently depletes the number and survival of p63-regulated sebocyte progenitor cells.

The expression of IGF-1, the most important pro-survival stimulus and mitogen of SGs, was increased in the basal and suprabasal layers of SGs of acne patients [7]. In normal skin, lGF-1 receptor (IGF1R) mRNA expression was most intense in the basal cells of the SG in immature sebocytes. Some weaker staining was present in mature fully differentiated sebocytes [119]. Expression was also detected in all cells of the infundibulum [123]. IGF-1 may thus promote infundibular keratinocyte proliferation (comedogenesis) in acne [124]. The pattern of IGF-1 and IGF1R expression suggests a critical role for IGF-1 as a sebaceous mitogen and morphogen [123]. IGF-1-deficient patients with Laron syndrome do not develop acne and other mTORC1-driven diseases of civilization [124, 125]. The expression pattern of the IGF-1/IGF1R system thus perfectly fits to the hyperproliferative cell layers of SGs and infundibular keratinocytes observed in acne patients [126, 127]. Importantly, p53 has been identified as a negative regulator of the *IGF1R* gene [128], which mediates increased IGF-1/mTORC1 signalling of puberty and Western diet (Fig. 1) [6, 22, 129]. Recent evidence underlines that the IGF-1 signalling axis and p53 genome protection pathways are tightly interconnected [130]. IGF-1/AKT/mTORC1 signalling also increases the anti-apoptotic regulator survivin [24, 25], which is upregulated in the skin of acne patients [26]. Survivin’s anti-apoptotic effects are mediated via inhibition of caspase 3 [131], which is the downstream effector caspase activated by TRAIL signalling [132]. FoxO3a, which is suppressed via IGF-1/AKT signalling [133], is an inducer of TRAIL expression (Fig. 1) [131]. Thus, p53-mediated inhibition of IGF-1 signalling will reduce survivin expression and its anti-apoptotic action in the pilosebaceous follicle. Furthermore, p53 and p53-mediated FoxO3a signalling increase pro-apoptotic TRAIL signalling.

Isotretinoin treatment of SEB-1 sebocytes induced G_1_ cell cycle arrest via upregulation of the cell cycle inhibitor p21 [134]. It is known that p53 uses cell cycle checkpoints to induce G_1_/S and G_2_/M cell cycle arrest [135, 136]. p21 (WAF1) was among the first p53 target genes that have been identified [137, 138].

mTORC1 signalling, which is increased in SGs of acne patients [10, 17], is negatively regulated by p53 [42, 116]. Deletion of p53 enhances mTORC1 activity by altering lysosomal dynamics of TSC2 and Rheb [139]. mTORC1 orchestrates the expression of SREBP1c and PPARγ [13–15], which play a crucial role in sebaceous lipogenesis, sebocyte differentiation, and sebum production [18, 19, 140–142].

**Fig. 2:**
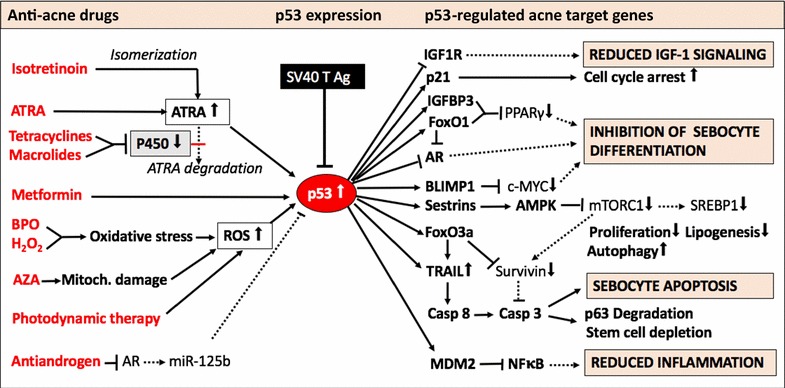
Synoptic illustration of p53-activating anti-acne therapies. Systemic isotretinoin (13-*cis* retinoic acid) via isomerization to *all*-*trans* retinoic acid (ATRA), tretinoin (ATRA), as well as cytochrome p450-inhibiting tetracyclines and macrolides all enhance ATRA-mediated upregulation of p53. Benzoyl peroxide (BPO) and hydrogen peroxide (H_2_O_2_) enhance p53 expression as well as a azelaic acid (AZA)-induced mitochondrial damage and photodynamic therapy, which generate reactive oxygen species (ROS). Activated p53 attenuates the expression of IGF-1 receptor (IGF1R) and of androgen receptor (AR). p53 activates expression of cell cycle inhibitor p21 and via upregulation of IGF binding protein-3 (IGFBP3) suppresses the transactivation of peroxisome proliferator-activated receptor-γ (PPARγ), which is important for sebocyte differentiation. Oxidative stress-responsive sestrins activate AMP kinase (AMPK), which inhibits mechanistic target of rapamycin complex 1 (mTORC1) downregulating anabolism, cell growth and sterol regulatory element binding protein 1c (SREBP1c)- and PPARγ-dependent lipogenesis. p53-mediated upregulation of FoxO1 expression inhibits AR, PPARγ, and SREBP1c, key transcription factors of sebaceous lipogenesis and sebocyte differentiation. p53-induced expression of FoxO3a and tumour necrosis factor-related apoptosis-inducing ligand (TRAIL) activate pro-apoptotic signalling with upregulation of caspase 8 (Casp8) and caspase 3 (Casp3), which execute apoptosis and promote p63 degradation. p53 increases the expression of the ubiquitin E3 ligase MDM2, which inhibits nuclear factor κB (NFκB), the key transcription factor for inflammatory cytokine expression. Anti-androgens attenuate AR-mediated expression of miRNA-125b, a key negative regulator of p53. Thus, p53 upregulation balances all pathological deviations observed in the sebaceous follicle of patients with acne vulgaris: increased proliferation, exaggerated lipogenesis, and inflammation. Note, that p53 is suppressed in SV40 immortalized sebocytes, because SV40 large T antigen physically inhibits p53

IGF binding protein-3 (IGFBP-3) is a nuclear regulator that binds to retinoid X receptor-α (RXRα) and several of its dimerization partners, including nuclear receptor Nur77 and PPARγ [143, 144]. RXRα-IGFBP3 interaction leads to modulation of the transcriptional activity of RXRα that is essential for mediating the effects of IGFBP3 on apoptosis [145]. In response to IGFBP3, the RXRα binding partner nuclear receptor Nur77 rapidly undergoes translocation from the nucleus to the mitochondria, initiating an apoptotic cascade resulting in caspase activation [146]. IGFBP3 attenuates the activation of PPARγ and inhibits adipocyte differentiation [147]. IGFBP3 interacted with PPARγ and inhibited PPARγ heterodimerization with RXRα [147]. Isotretinoin treatment of SEB-1 sebocytes resulted in a threefold over-expression of IGFBP3 [119]. Notably, IGFBP3 is a target gene of p53 [148]. Thus, p53-mediated induction of IGFBP3 gene expression inhibits mitogenic IGF-1 signalling (Fig. 2).

Taken together, pro-apoptotic isotretinoin-ATRA-p53 signalling induces a complex regulatory network that counteracts exaggerated IGF-1-AKT-mTORC1-mediated pro-survival signalling in acne vulgaris. Whereas isotretinoin-induced p53-TRAIL signalling is the desired effect promoting sebum suppression via sebocyte apoptosis, all adverse effects of the drug including teratogenicity can be explained by p53-mediated apoptosis of vulnerable ATRA-sensitive cells such as neuronal crest cells (Table 1) [149]. Intriguingly, hyper-activated p53 induced neural crest cell apoptosis in mice and craniofacial abnormalities resembling retinoid embryopathy [150, 151].

**Table 1:** p53-regulated target genes involved in isotretinoin’s mode of action

p53 target genes	Desired and adverse drug effects
Tumor necrosis factor-related apoptosis-inducing ligand, TRAIL (*TNFSF10*) upregulation	Sebocyte apoptosis: sebum suppression Meibomian cell apoptosis: dry eyes Neural crest cell apoptosis: teratogenicity Hypothalamic cell apoptosis: depression Intestinal cell apoptosis: inflammatory bowel disease
Insulin-like growth factor-1 receptor (*IGF1R*) suppression	Attenuated pro-survival and mitogenic signaling of IGF-1
Androgen receptor (*AR*) suppression	Reduced AR expression and miRNA-125b-mediated suppression of p53
IGF binding protein-3 (*IGFBP3*) upregulation	Enhanced pro-apoptotic signalling and suppressed PPAR*γ* signalling: attenuated lipogenesis
Cyclin-dependent kinase inhibitor 1A, p21 (*CDKN1A*) upregulation	G1/S cell cycle arrest: Suppression of comedogenesis and sebocyte proliferation
B lymphocyte-induced maturation protein 1 (BLIMP1) *(PRDM1*) upregulation	Increased BLIMP1-mediated c-Myc suppression reducing sebocyte differentiation
Sestrin 1 (*SESN1*) and sestrin 2 (*SESN2*) upregulation	Activation of AMPK resulting in mTORC1 and ACC inhibition: sebum suppression
Forkhead box O1 (*FOXO1*) upregulation	Suppression of AR, SREBP1c and PPAR*γ*: suppression of lipogenesis
Forkhead box O3a (*FOXO3A)* upregulation	Enhanced upregulation of TRAIL: enhancement of apoptosis
AMP-activated protein kinase (*PRKAA1*)	Increased expression of AMPK and AMPK-mediated inhibition of mTORC1
Aquaporin 3 (*AQP3*) upregulation	Increased aquaporin 3 expression: increased transepidermal water loss, dry skin, xerosis,
Aquaporin 4 (*AQP4)* upregulation	Increased aquaporin 4 expression increasing cerebrospinal fluid (risk of *pseudotumor cerebri*)
Apolipoprotein B100 (*APOB*) and apoB mRNA editing enzyme complex 1 (*APOBEC1*)	Increased hepatic synthesis of ApoB100: hypertriglyceridaemia with increased hepatic secretion of triglyceride-rich VLDL

## Anti-androgens

Antiandrogens play an important role in sebum suppression and acne therapy in female patients [152, 153]. Androgen receptor (AR)-mediated signalling contributes to sebocyte differentiation and maximization of sebaceous lipogenesis [154]. In hamster sebocytes, phosphorylation and thus activation of TOR was increased by the addition of testosterone in the presence of IGF-1 [154]. Furthermore, IGF-1 enhances adrenal and gonadal androgen synthesis and via activation of 5α-reductase promotes the conversion of testosterone to its ten times more potent AR-ligand dihydrotestosterone (DHT) [6]. Increased IGF-1 signalling in acne suppresses nuclear FoxO1 [8–10], which is a nuclear co-suppressor of AR [155], and thus increases AR-mediated target gene expression. Recently, p53 has been identified as transcriptional inducer of FOXO1 and PTEN [156], an important observation that confirms the role of p53 in regulating multiple signalling levels of IGF-1/IGF1R/PI3K/AKT/FoxO1 signalling. AR is regarded as a sensitive marker of sebaceous differentiation [157]. Androgens induce sebaceous differentiation in sebocytes expressing a stable functional AR. DHT up-regulated the expression of genes potentially related to sebocyte differentiation such as *MUC1/EMA*, *AQP3*, and *FADS2* [158]. Remarkably, *AR* is a direct target of p53 and is negatively regulated by p53 [159, 160]. This allows the conclusion that all p53-activating anti-acne agents attenuate AR signalling and thus exert anti-androgenic activity, which is further suppressed via classical anti-androgens such as cyproterone acetate (CPA).

c-Myc is a further important transcription factor promoting sebocyte differentiation [161, 162]. Interestingly, a functional interaction between c-Myc and p53 has been reported [163]. Expression of c-Myc significantly attenuated apoptosis and impaired the transcriptional activity of p53 on p21 [163]. c-Myc overexpression may antagonize the pro-apoptotic function of p53 [163]. Recent evidence indicates that c-Myc-induced SG differentiation is controlled by an AR/p53 axis [163]. c-Myc-induced SG differentiation was reduced in mice lacking a functional AR. In contrast, testosterone treatment or p53 deletion activated AR signalling and restored c-Myc-induced differentiation [164]. Recent studies have revealed that FoxO3a acts as an antagonist of c-Myc (Fig. 1) [165]. Thus, increased IGF-1-AKT signalling in acne via FoxO3a suppression may favour c-Myc-driven SG differentiation.

Anti-androgens with proven effects in the treamtment of acne are CPA, spironolactone and flutamide [152, 153]. These three major anti-androgens used for acne therapy are AR ligands that antagonize the actions of testosterone and DHT by competing for AR binding sites. Testosterone and DHT-mediated activation of AR induces the expression of miRNA-125b [166, 167]. Importantly, miRNA-125b is a highly conserved key suppressor of p53 [168–170]. The *MIR125B2* gene promoter exhibits four AR response elements pointing to close interaction between androgens and miRNA-125b expression [167]. Anti-androgens such as CPA or flutamide reduce AR-mediated expression of miRNA-125b [167], which increases p53 activity [167–170]. Remarkably, p53-dependent expression of the pro-apoptotic proteins TRAIL and death receptor 5 (DR5) increased by CPA treatment [171]. p53 suppresses the expression of AR, thus reduces AR signaling [159, 160]. Indeed, oral isotretinoin, which enhances p53 activity, has been demonstrated to reduce AR levels in the skin of isotretinoin-treated acne patients [172]. In this regard, isotretinoin and anti-androgens converge in p53-induced TRAIL-mediated sebocyte apoptosis and sebum suppression.

Androgen/AR-induced miRNA-125b not only targets p53 but also BLIMP1 [173]. p53 positively regulates BLIMP1 transcription [174]. BLIMP1 is a suppressor of c-Myc [175]. Anti-androgen treatment of acne via attenuation of miRNA-125b may thus increase the inhibitory effect of BLIMP1 on c-Myc thereby inhibiting sebocyte differentiation and sebaceous lipogenesis.

## Immortalized sebocytes

A huge number of experimental acne research has been performed with immortalized sebocytes such as the SZ95 or SEB-1 sebocyte cell lines, which are derived from human sebocytes transfected with the SV40 large T antigen [176, 177]. Via transfection of the HPV16-E6/7 oncoproteins, the immortalized human sebocyte cell line SEBO662 has been established [178]. It is believed that immortalized sebocyte culture models provide valuable insights into the development and management of acne [179, 180]. However, immortalized cell lines are not a suitable model to study acne nor the in vivo pharmacological action of anti-acne agents as immortalization abolishes p53 activity [179, 180]. Immortalization by SV40 large T antigen and oncogenic HPV16 proteins inactivates p53, pRB and SEN6 [180, 181].

The large T antigen of simian virus 40 (SV40) forms a specific complex with p53 [182, 183] and inhibits p53-mediated transcription [184] (Fig. 2). During evolutionary viral adaptation to host organisms, viruses have developed strategies to manipulate host cell p53 dependent pathways to facilitate viral survival via inhibition of host cell apoptosis [185]. SV40 T antigen-mediated p53 suppression apparently impairs ATRA-p53-induced sebocyte apoptosis. In fact, isotretinoin (10^−8^ − 10^−5^ M) did not affect externalized phosphatidylserine levels, DNA fragmentation, and lactate dehydrogenase cell release, despite increased caspase 3 levels [186]. Only, after addition of a further apoptosis-inducing agent (staurosporine) DNA fragmentation in SZ95 sebocytes was induced [186]. In B16F-10 melanoma cells, isotretinoin alone induced apoptosis associated with upregulated p53 expression [187]. Despite a multitude of studies with immortalized sebocytes, no data on the expression and regulation of p53, the guardian of the genome, have yet been reported.

## Conclusion

There is compelling evidence for the key role of p53 in sebocyte homeostasis. It can be concluded from translational evidence that currently available anti-acne agents have a common mode of action: the upregulation of p53 expression. p53 controls a web of critical genes related to acne pathogenesis such as AR, FoxO transcription factors, BLIMP1, and mTORC1 activity, that all play a key role in acne pathogenesis as well as pharmacological actions of anti-acne agents [188]. p53, the guardian of the genome, is a pivotal regulator for cell homeostasis. p53 controls most important cellular responses such as IGF-1 and AR signalling and via induction of MDM2 terminates p53-induced cellular responses via ubiquitination and proteasomal degradation of p53, FoxO1 and FoxO3a, respectively [189–191]. All these essential regulatory mechanisms are compromised in immortalized sebocytes via transfection with SV 40 large T antigen or HPV16-E6/7 oncoproteins. In contrast to the in vivo situation, p53 in acne patients is not artificially inactivated and still responsive to pharmacological targeting. We have to appreciate that acne is a pro-survival disease of the sebaceous follicle with increased IGF-1/AKT/mTORC1-survivin signalling [192]. Anti-acne agents such as retinoids, antibiotics, peroxides, azelaic acid, metformin and anti-androgens induce p53-mediated signalling and thus readjust the delicate p53-dependent balance between survival and death. Immortalized sebocytes with inactivated p53 transcription are thus a most critical and perhaps misleading model system to study p53-driven apoptotic signalling pathway in acne, which have recently excited the field of acne research [193].

## Abbreviations

BLIMP1: B-lymphocyte-induced nuclear maturation protein 1
AKT: Akt kinase (protein kinase B)
AMPK: AMP-responsive protein kinase
AR: androgen receptor
ATRA: *all*-*trans* retinoic acid
AZA: azelaic acid
BPO: benzoyl peroxide
CPA: cyproterone acetate
DHT: dihydrotestosterone
FoxO: Forkhead box O
HPV: human papilloma virus
IGF-1: insulin-like growth factor-1
IGFBP-3: IGF binding protein 3
IGF1R: IGF-1 receptor
MDM2: mouse double minute homolog 2
mTORC1: mechanistic target of rapamycin complex 1
NFκB: nuclear factor κB
PDT: photodynamic therapy
PI3K: phosphoinositide-3 kinase
PPAR: peroxisome proliferator-activated receptor
RB: retinoblastoma protein
RAR: retinoic acid receptor
ROS: reactive oxygen species
RXR: retinoid receptor
SG: sebaceous gland
S6K1: S6 kinase 1
SREBP: sterol regulatory element binding protein
SV40: simian virus 40
TRAIL: tumour necrosis factor-related apoptosis-inducing ligand
TSC2: tuberin
